# Optimized Design of Low-Carbon Mix Ratio for Non-Dominated Sorting Genetic Algorithm II Concrete Based on Genetic Algorithm-Improved Back Propagation

**DOI:** 10.3390/ma17164077

**Published:** 2024-08-16

**Authors:** Fan Zhang, Bo Wen, Ditao Niu, Anbang Li, Bingbing Guo

**Affiliations:** 1 Department of School of Civil Engineering, Xi’an University of Architecture and Technology, Xi’an 710055, China; zz7410666@163.com (F.Z.); niuditao@163.com (D.N.); lianbang@xauat.edu.cn (A.L.); guobingbing212@163.com (B.G.); 2 State Key Laboratory of Green Building in Western China, Xi’an University of Architecture and Technology, Xi’an 710055, China; 3 Key Laboratory of Structural Engineering and Seismic Education, Xi’an University of Architecture and Technology, Xi’an 710055, China

**Keywords:** machine learning, intensity prediction, BP, GA-BP, NSGA-II optimization

## Abstract

In order to achieve low-carbon optimization in the intelligent mix ratio design of concrete materials, this work first constructs a concrete mix ratio database and performs a statistical characteristics analysis. Secondly, it employs a standard back propagation (BP) and a genetic algorithm-improved BP (GA-BP) to predict the concrete mix ratio. The NSGA-II algorithm is then used to optimize the mix ratio. Finally, the method’s accuracy is validated through experiments. The study’s results indicate that the statistical characteristics of the concrete mix ratio data show a wide distribution range and good representativeness. Compared to the standard BP, the fitting accuracies of each GA-BP set are improved by 4.9%, 0.3%, 16.7%, and 4.6%, respectively. According to the Fast Non-Dominated Sorting Genetic Algorithm II (NSGA-II) optimization for meeting C50 concrete strength requirements, the optimal concrete mix ratio is as follows: cement 331.3 kg/m^3^, sand 639.4 kg/m^3^, stone 1039 kg/m^3^, fly ash 56 kg/m^3^, water 153 kg/m^3^, and water-reducing agent 0.632 kg/m^3^. The 28-day compressive strength, material cost, and carbon emissions show relative errors of 2.1%, 0.6%, and 2.9%, respectively. Compared with commercial concrete of the same strength grade, costs and carbon emissions are reduced by 7.2% and 15.9%, respectively. The methodology used in this study not only significantly improves the accuracy of concrete design but also considers the carbon emissions involved in the concrete preparation process, reflecting the strength, economic, and environmental impacts of material design. Practitioners are encouraged to explore integrated low-carbon research that spans from material selection to structural optimization.

## 1. Introduction

Currently, concrete, as a primary building material, is widely used due to its easy availability of raw materials, excellent performance, ease of preparation, and various other advantages. With the shift in building structures from short, low, and shallow to long, high, and deep, more stringent requirements have been imposed on the performance of building materials and structural design [1]. In light of the introduction of relevant environmental protection policies and the proposal of the “dual carbon” goals, reducing the carbon emissions of buildings has become a prominent research topic. Numerous civil engineering researchers have begun investigating low-carbon building materials, the recycling and high-value utilization of solid waste materials, and CO₂ curing, among other areas [2,3,4]. For environmental impact assessment, it is now common practice to use life-cycle evaluation methods to refine the concrete construction process and calculate total process carbon emissions using the carbon emission factor method, thereby assessing its environmental impact [5,6]. As the first step in engineering construction, the design of the mixing ratio needs to consider aspects such as safety, durability, workability, economy, and environmental impact [7]. Both domestic and international architectural designers primarily follow the ordinary concrete proportion design regulations (JGJ 55-2011). Through trial calculations, trial mixes, and other steps, the content of the various components of concrete is determined. While the material design process has become more refined, traditional design methods still waste manpower and materials. With the advancement of computer functions and algorithms, researchers have begun integrating machine learning with engineering practice to achieve more accurate predictions [8,9]. Shiqi Wang [10] and others discussed the research progress of applying artificial intelligence (AI) in the construction field, highlighting the current advantages and disadvantages. Their study shows that AI has higher accuracy for the nonlinear prediction of structural or material properties. However, due to the limitations of the algorithms themselves, inaccurate calculations may occur. Therefore, it is crucial to choose a suitable algorithm based on the specific problem at hand.

For concrete proportion design, Zhang JK et al. [11] addressed the issues of large volume and complexity in traditional proportion trial calculations by integrating machine learning into concrete proportion prediction. They developed a rubber concrete strength prediction model using the limit learning machine algorithm. The results indicated that this model, compared to traditional algorithmic models, demonstrated high accuracy and generalization ability. Hussain, F et al. [12] applied intelligent optimization algorithms to lightweight aggregate concrete design. Using 420 sets of data, they compared five intelligent algorithms—Support Vector Machines (SVMs), Artificial Neural Networks (ANNs), Decision Trees (DTs), Gaussian Process Regression (GPR), and Extreme Gradient Boosting Trees (XGBoost)—to evaluate their performance. The study found that the Gaussian regression machine model had the highest prediction accuracy. Siddique, R [13] used neural network techniques to predict the 28-day compressive strength of self-compacting concrete with admixtures and evaluated the algorithmic model. Zhang W et al. [14] compared various machine learning techniques, including Random Forest, Support Vector Machine, and neural networks, to predict the residual strength of concrete and identified the best model for their study. Al-Shamiri, AK et al. [15] employed regularized machine learning to develop a compressive strength prediction model for high-performance concrete, demonstrating that their proposed model achieved high accuracy. Relevant studies have shown that the optimization and improvement of algorithms can enhance their optimization-seeking capabilities [16,17]. Genetic algorithms, known for their strong search abilities and capability to handle complex problems, have been used to improve neural network algorithms [18].

In addition, AI demonstrates significant advantages in concrete crack detection and high-performance concrete performance prediction. Ahcene Arbaoui et al. [19,20] utilized convolutional neural networks (CNNs) for detecting cracks in concrete structures, employing deep learning to achieve automatic crack identification. The study results indicated that this method offers high accuracy. Naimul Islam [21] used a deep learning model to predict the compressive strength of high-performance concrete, comparing four deep learning models—BiLSTM, CNN, GRU, and LSTM. The results showed that the GRU model provided the highest accuracy.

Domestic and international research on the optimization of concrete ratios aims at achieving multiple performance goals or optimizing preparation parameters. Jiajie Li et al. [22] conducted orthogonal tests on the preparation conditions of bulk solid waste and steel slag. Their study established a solid foundation for the development of carbonation materials from steel slag tailings. Gong Y et al. [23] utilized the surface response method to design the ratio of rubberized basalt fiber concrete. Their experiments determined the optimal preparation parameters. Analysis showed that rubber content significantly affects the compressive strength of concrete, while fiber content has a notable impact on the flexural strength. The optimal preparation parameters were found to be a water–cement ratio of 0.39, fiber content of 4.56 kg/m^3^, and a rubber dosage of 10%. With the gradual maturation of computer algorithms, various optimization algorithms have increasingly been applied to practical engineering research. Wang Xiao-Yong [24] utilized genetic algorithms to determine the optimal amount of limestone powder admixture in concrete, aiming to reduce CO_2_ emissions. Emadaldin Mohammadi Golafshani [25] collected extensive data and used the BBP geographic planning algorithm to predict concrete properties. The developed algorithmic model, incorporating relevant constraints, determined the optimal mix ratio for silica fume concrete. Chen B [26] et al. aimed to design highly durable concrete using a multi-objective optimization framework that combined random forest and the non-dominated genetic algorithm. This approach effectively predicted concrete durability and optimized the mix ratio. The study found that the prediction model had high accuracy (Pearson correlation coefficient of 0.95), and the concrete optimized by NSGA-II exhibited greatly improved durability, with a cost of only 376.77 yuan per unit volume. Xia Jingliang [27] et al. used the surface response method to design concrete proportions with limestone powder and analyzed the impact of various factors on concrete performance through central composite design. They proposed an optimal proportion using multi-objective optimization. Chen Hongyu [28] et al. developed a non-dominated optimization framework based on the Random Forest Algorithm, Least Squares Support Vector Machine (LSSVM), and Elite Strategy Genetic Algorithm (NSGA-II) to prepare high-performance concrete with cost efficiency. The study showed that the LSSVM-NSGA-II algorithm had better optimization results, improving permeability and frost resistance by 30.71% and 3.17%, respectively, while reducing costs by 1.84%. This intelligent optimization algorithm offers valuable guidance for concrete proportion selection. The NSGA-II algorithm is known for its good convergence and global optimization ability, short computation time, and strong scalability. It is widely used in multi-objective optimization problems [29].

In summary, to address the complicated trial calculation and mixing process of traditional ordinary concrete proportions, more people are combining artificial intelligence with practical engineering. Researchers are using machine learning, deep learning, and other methods for concrete performance prediction, as well as optimizing concrete through genetic algorithms or genetic algorithms with elite strategies. However, the applied research of artificial intelligence has shown a tendency to be one-sided. Most studies focus either on concrete performance prediction using AI algorithms or on single-objective or multi-objective optimization using algorithms without a complete integration of AI for both concrete prediction and optimization. Regarding prediction algorithms, there are numerous algorithms available, making it challenging to reasonably choose and improve them. For optimization, most researchers concentrate on balancing performance and cost control objectives, with few taking environmental impact into consideration as an optimization goal. Based on this, the study uses carbon emissions as the optimization index for the optimal design of the mix ratio. First, a model for concrete mix ratio and compressive strength was established using machine learning. The 28-day compressive strength of concrete was predicted using standard BP and GA-BP models, with evaluation conducted through mean absolute error (MAE), mean squared error (MSE), root mean squared error (RMSE), and mean absolute percentage error (MAPE). Building on this, the 28-day compressive strength prediction model was used as the objective function, while the objective function for concrete carbon emissions was constructed based on the carbon emission factor method. Concrete cost was selected as the third objective function. Specifications and project requirements for material dosage were used as constraints. The fast non-dominated sorting algorithm (NSGA-II) was employed to find the optimal cost-effective combination of proportion parameters. The optimal cost-effective combination was then obtained through testing. Finally, the measured and predicted values are compared and analyzed to verify the accuracy of the optimization design method.

## 2. Theoretical Foundation

### 2.1. BP Fundamentals

**Figure 1:**
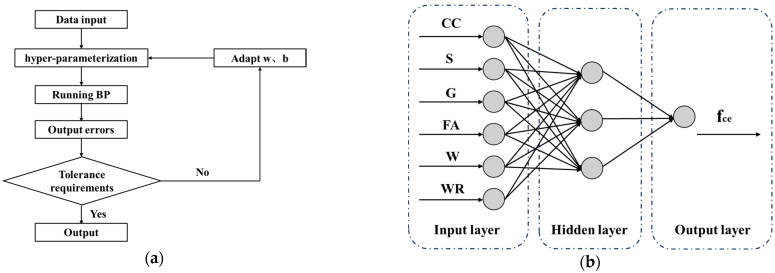
Concrete BP modeling process and model building. (**a**) BP Work flow; (**b**) BP model construction.

The BP workflow and modeling process are illustrated in Figure 1a. As shown, the program design begins with importing the dataset, followed by dividing it into a training set and a test set. Next, the model is established, and parameters such as the number of training iterations, learning rate, and other grid parameters are set. The model’s output is then evaluated using error indicators to determine if it meets the required accuracy. If the error requirements are met, the model output is accepted. Due to the numerous factors influencing concrete performance predictions, linear models are often insufficient. Instead, neural network fitting provides a more accurate black-box model. In this paper, standard BP learning was performed using the mathematical software Matlab R2022a, published by MathWorks, Inc. Figure 1b illustrates the working process of the network’s input layer, hidden layer, and output layer. The input layer comprises six indicators: Cement (CC), Sand (S), Gravel (G), Fly Ash (FA), Water (W), and Water Reducer (WR). The hidden layer employs the sigmoid activation function, and the output layer predicts the 28-day compressive strength of the concrete (fce).

In this study, Equation (1) is used to determine the optimal number of neurons in the hidden layer of the BP neural network. With m = 1 and n = 6, and *a* being an arbitrary number, the number of neurons is optimized through trial and error based on the output error metrics. The specific results, including error metrics graphs from Section 4.1, lead to the conclusion that the optimal number of neurons in the hidden layer is 12, with a = 10 in the present study.

(1) $$l=\sqrt{m+n}+a$$

where m is the number of output neurons; n is the number of input layer neurons; and a is a constant between 1 and 12.

### 2.2. GA-BP Theory

The standard BP network can handle simple nonlinear mapping relationships, but it is prone to getting stuck in local minima. Genetic algorithms (GAs) offer superior global optimization capabilities, addressing the limitations of the standard BP neural network’s search ability. By optimizing the thresholds and weights of the neural network, GAs enhance the BP network’s computation and optimization performance. This approach helps overcome the issue of local minima and improves the network’s prediction accuracy [30,31,32].

### 2.3. NSGA-II Arithmetic

**Figure 2:**
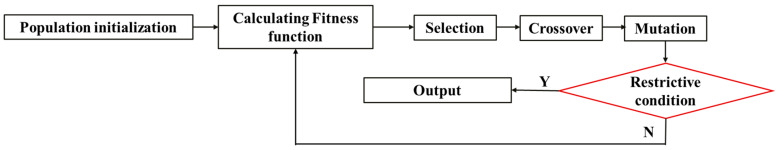
Genetic algorithm flow.

The basic steps of the genetic algorithm are illustrated in Figure 2 below, which highlights the construction of the objective function, the selection of hyperparameters, and the establishment of constraints.

The Fast Non-Dominated Sorting Genetic Algorithm II (NSGA-II) is an advanced version of the GA. Its core principle is to find optimal solutions within the solution space by simulating biological evolution processes such as selection, crossover, and mutation. Compared to traditional genetic algorithms, NSGA-II offers higher complexity, greater applicability, and faster convergence speeds. It enhances the optimization capabilities of GAs by incorporating an elite retention strategy, which helps achieve more efficient and effective optimization results.

### 2.4. Multi-Objective Optimization Model Building

**Figure 3:**
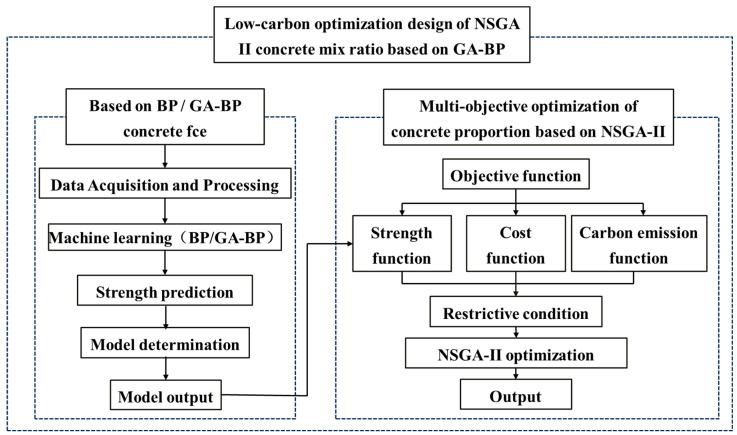
Technology roadmap.

Research indicates a nonlinear relationship between the dosage of each concrete component and its strength, necessitating numerous trial calculations during concrete proportion design to determine the precise functional form [33,34,35]. Neural network models can replace traditional mathematical functions to capture this complex relationship. Additionally, the output of the neural network model can serve as the objective function for the NSGA-II algorithm, enabling more accurate optimization. The specific workflow of the NSGA-II hybrid model based on GA-BP is illustrated in Figure 3.

The accuracy of BP and GA-BP prediction models for concrete with different mix ratios is evaluated based on relevant performance metrics. After comparative analysis, the machine learning model with the higher accuracy is selected. The NSGA-II algorithm is then employed to achieve three-objective optimization, focusing on work performance, cost, and carbon emissions. This approach determines the optimal mix ratio parameters that balance these three objectives. Finally, the reasonableness of the optimization framework is verified through experimental validation.

### 2.5. Algorithmic Implementation

**Figure 4:**
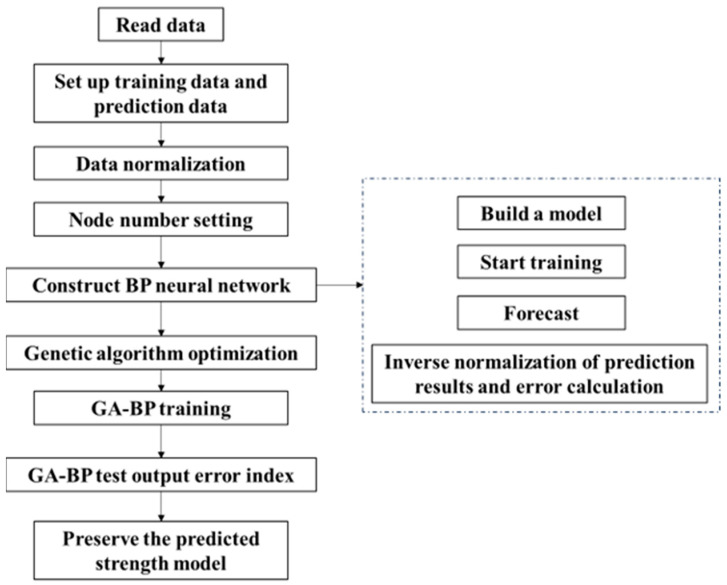
Genetic algorithm improvement process.

The algorithm implementation utilized selected MATLAB R2022a tools. Figure 4 illustrates the genetic algorithm improvement process. Initially, the fit ratio dataset is imported and divided into input and output components. To mitigate the impact of different scales, the data are normalized. After optimization, the program applies anti-normalization to derive accurate prediction values. The neural network model’s accuracy relies on determining the number of neurons and hidden layers, with the number of neurons in the hidden layer previously established. The BP (Backpropagation) model is used to output the relevant errors. The genetic algorithm improves this process by enhancing the weights and thresholds between the layers. It performs optimization processing by calling the genetic algorithm to optimize the BP model. Finally, the optimized error index is outputted, allowing for the comparison of the two schemes. The optimized model is then saved and used as the optimization objective function.

**Figure 5:**
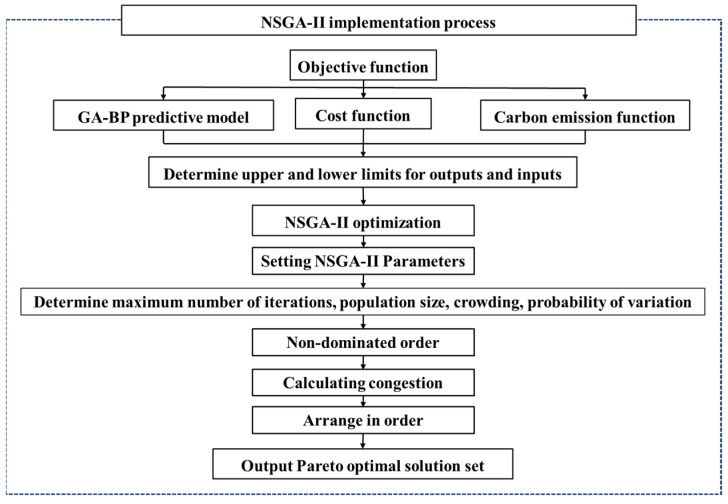
NSGA-II optimization process.

Figure 5 illustrates the implementation of the NSGA-II algorithm. This involves the following steps:

1. Objective Function Development: Construct the objective functions for intensity, cost, and carbon emissions.
2. Variable Limits Definition: Establish the upper and lower bounds for output and input variables according to the specifications.
3. Optimization Execution: Execute the optimization using the NSGA-II program, which requires setting various parameters, including maximum number of iterations, population size, degree of crowding, and mutation probability.
4. Non-Dominated Sorting: Perform non-dominated sorting to classify solutions based on dominance.
5. Crowding Distance Calculation: Compute the crowding distance for each solution to ensure diversity.
6. Solution Sorting and Output: Sort the solutions and generate the Pareto optimal solution set.

This methodology ensures that the NSGA-II algorithm effectively optimizes the trade-offs between intensity, cost, and carbon emissions.

## 3. Concrete Strength Prediction Study

### 3.1. Sample Statistics

**Figure 6:**
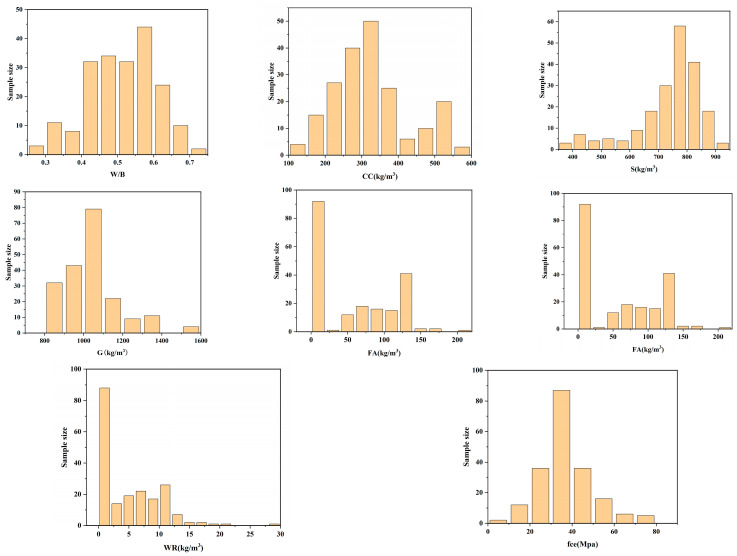
Distribution of samples for each feature.

To assess the impact of individual mix ratio components on the mechanical properties of concrete, this study selects six parameters: water-binder ratio (W/B), cement content (CC), sand (S), gravel (G), fly ash (FA), water (W), and water reducer (WR). The compressive strength of concrete (fce) is chosen as the model output variable for the neural network. Data from the past five years, including both experimental data from our group and literature summaries [36,37,38,39,40], amounting to a total of 200 experimental samples, are utilized. Carbon emissions for each sample are calculated using the carbon emission factor method [41] and are denoted as CE. Table 1 presents the sample distribution, and Figure 6 shows the distribution of the samples. Table 2 provides the statistical characteristics of the data, indicating that the selected data range is broad and encompasses the strength requirements of current concrete applications.

**Figure 7:**
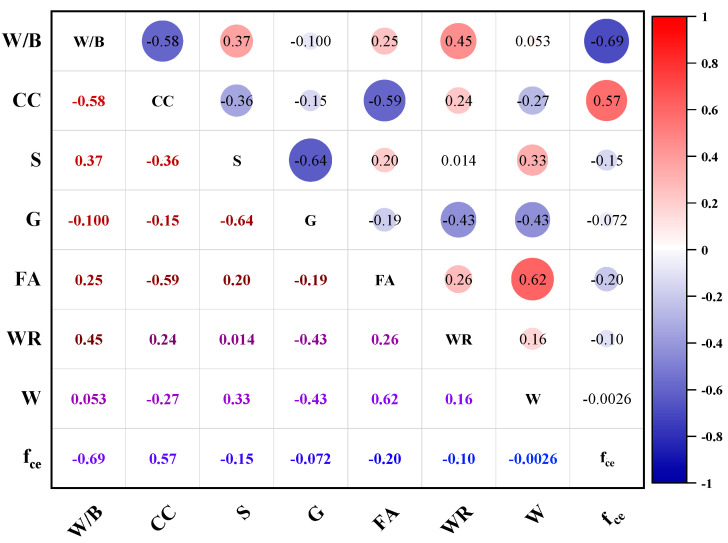
Pearson’s correlation coefficient for each variable.

The Pearson correlation coefficient can measure the strength and direction of linear correlation between two variables, and its value ranges from −1 to 1; 1 indicates complete positive correlation, −1 indicates complete negative correlation, and 0 indicates no linear correlation. Plotting the Pearson correlation coefficient heat matrix of each factor, specifically as shown in Figure 7, the correlation relationship between fce and each indicator can be seen, and the output of the Pearson is less than 1.

The correlation coefficients between the water–cement ratio, cement, and fly ash with the compressive strength of concrete are 0.69, 0.57, and 0.2, respectively. In contrast, the correlation coefficients for the remaining variables are relatively low. This indicates that the relationship between fce (compressive strength) and the seven input variables is not a simple multivariate linear relationship. Linear fitting cannot accurately establish the relationship between each variable and fce. Therefore, a nonlinear mapping relationship between fce and each input variable needs to be established through machine learning.

After analyzing the data using Pearson’s correlation coefficient, machine learning was performed with the BP and GA-BP algorithms to build the objective function. Eighty percent of the total sample set was used for training, while twenty percent was reserved for testing. The BPNN model was implemented using NET. Additionally, 10 sets of data were selected for prediction. Figure 4 shows that the water–cement ratio has the most significant impact on concrete strength. Therefore, the specific dosages of cement and water will be examined to determine their effect on compressive strength. For clarity, the six factors are renamed as follows: cement dosage (X1), sand dosage (X2), crushed stone dosage (X3), mass fraction of fly ash (X4), water content (X5), and water reducer mass fraction (X6).

### 3.2. Data Normalization

The training process of neural networks typically relies on optimization algorithms such as gradient descent. Due to the large differences in the magnitude of the input sample values, it is necessary to preprocess the data to improve training accuracy and stability, as well as to enhance the model’s generalization ability. To achieve this, the input data are normalized using Equation (2), which scales the data to fall within the range [0, 1].

(2) $${\bar{X}}_{i}=\frac{X_{i\;}-X_{min}}{X_{max}-X_{min}}$$

where ${\bar{X}}_{i}$ denotes the normalized value, $X_{i}$ the ith input value, and $X_{max}$, $X_{min}$ are the maximum and minimum values of the data on the neuron, respectively.

### 3.3. Evaluation Indicators

To evaluate the prediction results accurately and efficiently, the mean absolute error (MAE), mean square error (MSE), root mean square error (RMSE), mean absolute percentage error (MAPE), and correlation coefficient (R) are used to assess the accuracy of both the BP model and the BP-GA model. MAE reflects the actual prediction error, while MSE indicates the extent of deviation between the predicted and actual values. RMSE measures the deviation between the predicted and actual values, and MAPE is an index used to assess the accuracy of the prediction model, effectively eliminating issues caused by error offsetting. Finally, R is used to evaluate the degree of fit between the predicted and actual values [42].

(3) $$MAE=\frac{1}{N}\sum_{i=1}^{N}\left|E_{i}-P_{i}\right|$$

(4) $$MSE=\frac{1}{N}\sum_{i=1}^{N}{\left(E_{i}-P_{i}\right)}^{2}$$

(5) $$RMSE=\sqrt{\frac{1}{N}\sum_{i=1}^{N}{\left(E_{i}-P_{i}\right)}^{2}}$$

(6) $$MAPE=\frac{\left|E_{i}-P_{i}\right|}{\left|E_{i}\right|}\times 100\%$$

(7) $$R=\sqrt{1-\frac{\sum_{i=1}^{n}{\left(E_{i}-P_{i}\right)}^{2}}{\sum_{i=1}^{n}{\left(E_{i}-P_{m}\right)}^{2}}}$$

where $E_{i}$ denotes the ith sample value test value; $P_{m}$ denotes the average of the sample model output values; and $P_{i}$ denotes the model predicted value for the *i*th sample.

## 4. Predictive Modeling Results and Analysis

### 4.1. Different Model Fits

**Figure 8:**
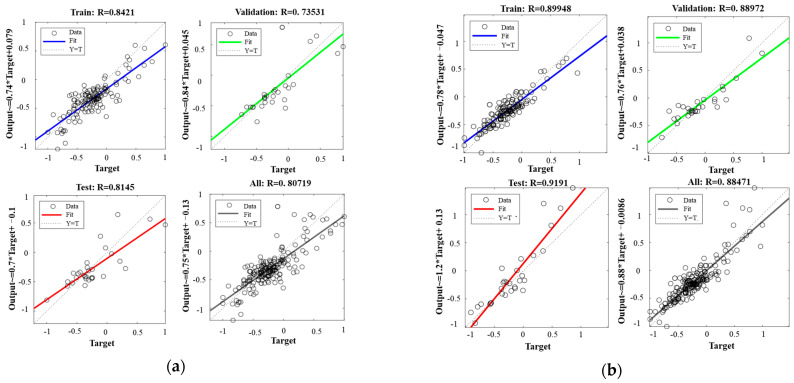
Comparison of different algorithms. (**a**) Standard neural network fit; (**b**) GA-BP fit.

Figure 8 displays the training set, validation set, test set, and overall prediction fitting results for both algorithms. The Pearson correlation coefficients for the standard BP model are 0.8421, 0.7353, 0.8145, and 0.8072 for the training set, validation set, test set, and all sets, respectively. For the GA-BP model, the correlation coefficients are 0.8995, 0.8897, 0.9191, and 0.8847 for the same sets. Compared to the standard BP model, the GA-BP model shows an improvement in fitting accuracy of 6.8%, 21.0%, 12.8%, and 9.6% for each set, respectively. The correlation coefficients for both networks are greater than 0.8, indicating good fitting accuracy. However, the GA-BP model demonstrates better performance compared to the standard BP model.

### 4.2. Comparison of Model Evaluation Indicators

**Figure 9:**
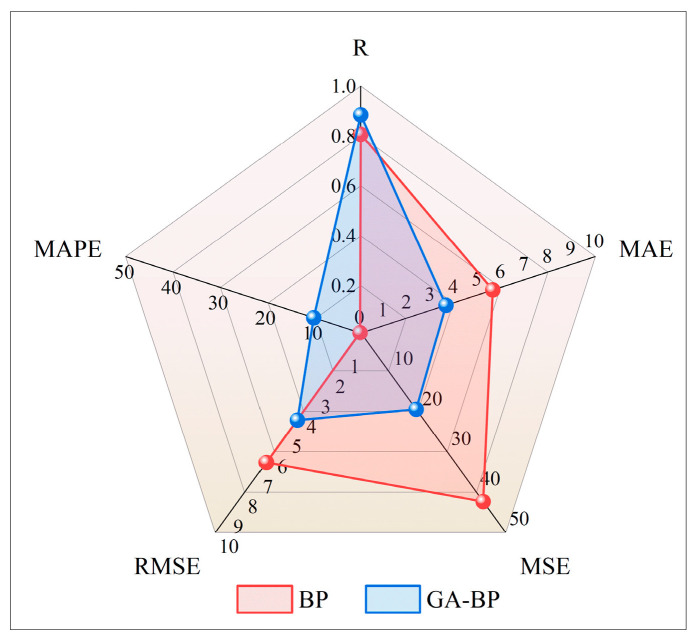
Comparison of different algorithms for each evaluation index.

**Figure 10:**
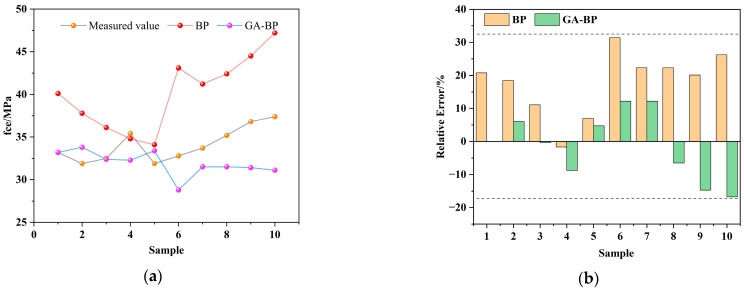
Comparison of prediction and comparison of errors. (**a**) Comparison of predicted and real values; (**b**) comparison of error.

The comparison results of BP neural network and GA-BP predictions are shown in Figure 9, while the evaluation index comparisons are presented in Figure 10. From Table 3, it can be observed that, compared to the standard BP model, the GA-BP model achieves a reduction in the MAE by 35.45%, the MSE by 54.76%, the RMSE by 32.74%, and the MAPE by 37.97%. These improvements indicate that the genetic algorithm significantly enhances the accuracy of the neural network predictions by optimizing the model parameters.

### 4.3. Analysis of Projected Results

The study investigates the concrete mix ratio using both BP neural networks and GA-BP networks. A prediction model for the 28-day compressive strength of concrete was established using 200 sets of data as training samples, with an additional 10 sets of mix ratio data used for prediction. The 28-day compressive strength test data and the corresponding prediction results are presented in Table 4. Figure 10a shows the comparison between the actual and predicted values, while Figure 10b illustrates the error comparison.

From Table 4 and Figure 10, it is evident that the compressive strength predicted by the optimized BP network is closer to the actual values, with errors controlled within ±17%. In contrast, the BP neural network shows larger errors, with some exceeding 20%. This indicates that the neural network optimized by the genetic algorithm exhibits better stability and accuracy. Therefore, due to its superior optimization capability, the GA-BP algorithm is chosen as the foundation for the concrete strength prediction model.

## 5. Genetic Algorithm to Optimize Concrete Ratio

### 5.1. Establishment of the Objective Function

To meet the strength requirements of concrete in practical applications while achieving the goals of low cost and minimal carbon emissions, the 28-day compressive strength of concrete, cost, and carbon emissions were set as the optimization objectives.

1. The 28d compressive strength function of concrete based on GA-BP

The compressive strength function utilizes the ratio strength prediction model trained by machine learning in MATLAB R2020a software, represented by the Max g1 function.

Max g1 = GA-BP (X1, X2, X3, ……, X6)(8)

where X1, X2……X6 corresponds to the composition of the concrete.

2. Construction of cost and carbon emission function

To account for the carbon emissions associated with different components of concrete production, the Construction Carbon Emission Calculation Standard GB/T 51366-2019 was used for calculation. Carbon emissions for various raw materials were determined using the carbon emission factor method. The market unit prices for the concrete mix ratio parameters were established based on the project’s actual conditions and are summarized in Table 4. Additionally, the carbon emission factors for the components were determined according to relevant standards and literature, as summarized in Table 5 [43,44,45].

The cost and carbon emission functions are as follows:

(9) $${Min\;C}_{T}=\sum X_{i}\cdot P_{i}$$

(10) $${Min\;E}_{T}=\sum X_{i}\cdot {EF}_{i}$$

where $C_{T}$ is the total cost of preparing concrete (CNY); $E_{T}$ is the carbon emission of preparing concrete (kg CO_2_e); $X_{i}$ is the amount of the ith type of concrete component (kg); $P_{i}$ is the unit price of the ith type of concrete component (CNY kg^−1^); and ${EF}_{i}$ is the carbon emission factor of the ith type of concrete component.

### 5.2. Establishment of the Scope of Constraints

To ensure a reasonable and accurate prediction and based on existing high-level literature and the project’s actual needs, the following parameters were selected for the concrete mix design: P·O42.5 cement for the binder, limestone crushed stone for the aggregate, and additional admixtures such as fly ash and water-reducing agents to enhance concrete strength. The specifications and engineering requirements, along with relevant data from our group, were used to determine a reasonable range for the mix ratio values [46]. Specifically, the ranges are as follows: water–cement ratio of 0.28 to 0.4, cement dosage of 330 to 450 kg/m^3^, sand dosage of 620 to 860 kg/m^3^, crushed stone dosage of 1030 to 1150 kg/m^3^, fly ash dosage of 56 to 98 kg/m^3^, and high-efficiency water-reducing agent dosage of 0.6% to 1.9%. The general form of these constraints is as follows:

(11) $$a_{imin}\;<{\;x}_{i}\;<{\;a}_{imax}$$

where $x_{i}$ denotes the content of each component in concrete, and $a_{imin}$ and $a_{imax}$ denote the upper and lower limits of each component in concrete, respectively.

Determine the concrete specific constraints for each material based on Equation (12), as follows:

(12) $${330\;\leq \;x}_{1\;}\leq \;450\;620{\;\;\leq \;x}_{2}\;\leq \;860\;1030\;{\leq \;x}_{3}\;\leq \;1150\;56\;{\leq \;x}_{4}\;\leq 98\;0.28\;\leq \;\frac{x_{5}}{x_{1}+x_{4}+x_{5}}\;\leq 0.4\;0.7\;\leq \;x_{6}\;\leq \;1.9$$

### 5.3. Three-Objective Optimization and Analysis of Elite Genetic Algorithm Based on GA-BP

1. GA-BP-based elite genetic algorithm for three-objective optimization

Based on the original data, three objective functions are selected, and constraints are determined accordingly. Using the NSGA-II algorithm, the maximum number of iterations is set to 60, the population size is 40, the degree of crowding is 0.8, the percentage of variance is 0.8, and the probability of variance is 0.03. The objectives include minimizing the total cost of compressive strength of the concrete, material costs, and carbon emissions. Optimization is performed using NSGA-II, resulting in the optimal 30 sets of fitment ratios and output values after 40 iterations. The results for the 40 sets of fitment ratios that meet the conditions are shown in Table 6.

**Figure 11:**
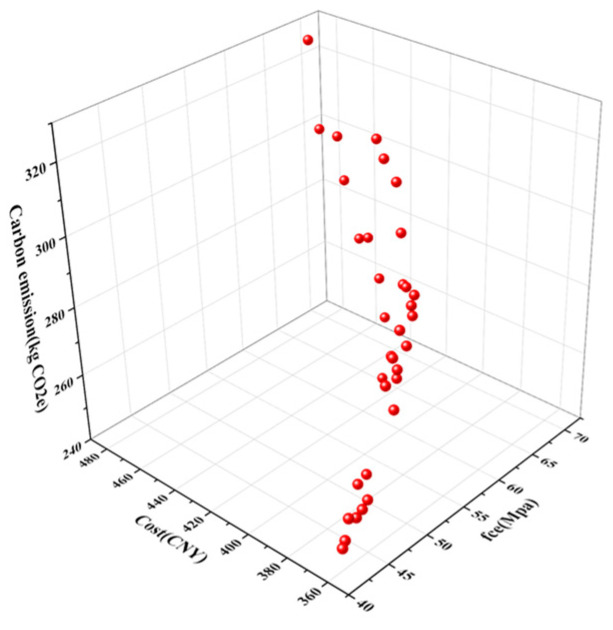
Three-objective optimization solution set.

As shown in Figure 11, both the cost of concrete and carbon emissions generally increase with higher compressive strength. When the compressive strength exceeds 60 MPa, the total cost of concrete rises more significantly. This increase is primarily due to the combined effect of material costs and carbon emission costs. Additionally, for concrete with compressive strengths ranging from 42.7 MPa to 68 MPa, the material costs range from 363 to 477 CNY, and carbon emissions vary between 244 and 328 kg CO_2_e. In a case study involving the exterior frame columns of the 40th to 54th floors of the first tallest building in Northwest China, located in Xi’an, Shaanxi Province, C50 concrete was used. To meet the minimum total cost requirement of 385.5 CNY, the concrete must have a 28-day compressive strength of 51.5 MPa and carbon emissions of 274 kg CO_2_e. At this optimal point, the material quantities per cubic meter of concrete are 378.2 kg of cement, 630.8 kg of sand, 1076 kg of crushed stone, 59 kg of fly ash, 153 kg of water, and 0.64 kg of water reducer.

2. Optimal Solution Verification

To further validate the accuracy of the optimization model, a 28-day compressive strength test was conducted on the concrete mix with the lowest total cost. The test used P.O 42.5 grade ordinary Portland cement produced by Shaanxi Qinling Cement Co., Ltd. in Xi’an, with an apparent density of 3.0 g/cm^3^. The main chemical components are listed in Table 7. The test also used Grade II fly ash developed and produced by Hancheng Datang Shenglong Technology Industrial Co., Ltd. (Hancheng, China), with an apparent density of 2.35 g/cm^3^. The coarse aggregate in the concrete consisted of high-quality shale gravel from Jingyang Mountain, with a particle size of 5–20 mm, an apparent material density of approximately 2.65 g/cm^3^, and a bulk apparent density of approximately 1.45 g/cm^3^. Laboratory tap water was used for the mix. The test was conducted according to the “Standard Test Methods for Physical and Mechanical Properties of Concrete” [47] using the mix proportions optimized by the model. The test results are shown in Table 8.

As shown in Table 8, the relative error between the predicted and experimental values for the 28-day compressive strength of concrete is only 2.1%. The relative error for material cost is 0.6%, and the relative error for carbon emissions is 2.9%. These results indicate that the optimized algorithm provides high prediction accuracy for concrete performance, cost, and environmental impact. The accuracy of the machine learning model is attributed to the effective capture of the nonlinear relationship between concrete strength and its proportions by the genetic algorithm. Additionally, the reliability of the NSGA-II algorithm contributes to this precision. The combination of neural networks with genetic algorithms has demonstrated that it can achieve accurate optimization, validating the effectiveness of the algorithm. Table 9 shows the comparison of each cover of concrete designed by this program with commercial concrete. As can be seen from Table 8, compared with ordinary commercial concrete, the 28d compressive strength of concrete designed by this program meets the strength requirements; in addition, its cost and carbon emissions are reduced by 7.2% and 15.9%, respectively, and the concrete designed by this method meets the strength of the basis and has better economic and environmental performance.

## 6. Conclusions

1. The concrete mix ratio is used as the input parameter, with the 28-day compressive strength as the output value. Using 200 datasets for training both the standard BP and GA-BP models, the simulation results indicate that the GA-optimized BP neural network provides superior prediction performance. Specifically, the average absolute error of the GA-BP network decreased by 35.45%, the mean square error by 54.76%, the root mean square error by 32.74%, and the average absolute percentage error by 37.97% compared to the standard BP network.
2. To reduce calculation complexity and enhance comprehensiveness, the carbon emissions generated during the concrete preparation process are considered. Using the carbon emission factor method, carbon emissions are modeled as a function of the mix ratio. Additionally, an objective function combining the mix ratio, total cost, and 28-day compressive strength is constructed, with constraints determined by relevant specifications and data. Optimized using the NSGA-II algorithm, the lowest total cost mix ratio for C50 concrete is found to be 331.3 kg/m^3^ of cement, 639.4 kg/m^3^ of sand, 1039 kg/m^3^ of crushed stone, 56 kg/m^3^ of fly ash, 153 kg/m^3^ of water, and 0.632 kg/m^3^ of water-reducing agent.
3. Comparing the measured indexes with the optimized values reveals that the relative errors between the measured and actual values for concrete’s 28-day compressive strength, material cost, and carbon emissions are 2.1%, 0.6%, and 2.9%, respectively. This indicates that the NSGA-II optimization algorithm combined with the GA-BP model achieves high accuracy and intelligence in optimizing concrete proportions, making the research results a valuable reference for existing projects.
4. Compared to commercial concrete of the same strength class, the concrete designed using this method not only meets the required strength but also offers superior economic and environmental benefits. Specifically, its carbon emissions are significantly reduced, achieving a 15.9% reduction compared to commercial concrete.
5. This study applies artificial intelligence to the optimization of low-carbon concrete mix proportions, and the research approach and methodology are presented in a systematic manner. However, there are some limitations: the dataset used is relatively small due to time constraints, but it can be expanded to improve the model’s accuracy in the future. Additionally, since the low-carbon optimization of mix ratios is only at the initial stage of energy saving and carbon reduction in the construction field, further research can explore integrated low-carbon optimization from materials to structures.

## Figures and Tables

**Table 1:** Sample data for plain concrete.

No.	W/B	CC kg/m^3^	S kg/m^3^	G kg/m^3^	FA kg/m^3^	W kg/m^3^	WR	fce MPa	Cost CNY	CE kg CO_2_e/m^3^
1	0.63	201	752	1276	61	165.06	1.18	16.8	349	153
2	0.68	186	800	1241	56	164.56	1.18	12.8	344	142
3	0.58	218	703	1307	66	164.72	1.18	19	355	165
⋮	⋮	⋮	⋮	⋮	⋮	⋮	⋮	⋮	⋮	⋮
198	0.57	238	752	1128	122	205	7.9	33.2	405	180
199	0.57	204	752	1128	163	163	8.1	31.9	406	156
200	0.57	170	752	1128	204	204	8.2	32.5	407	131

**Table 2:** Statistical characteristics of each parameter.

Type	Sample Size	Min	Max	AVG	SD	Median
W/B	200	0.27	0.7	0.50714	0.9637	0.51
CC		144	592	327.092	103.952	312.85
S		388	901.8	736.1065	113.478	768.5
G		801	1555	1034.956	143.619	1014
FA		0	204	54.554	55.58	54
W		82.8	293.304	189.076	37.103	186.59
WR		0	28.2	4.83	5.002	3
f_ce_		9.55	79.99	36.84	12.5387	35.98

**Table 3:** Indicators for evaluating forecast results.

Type	R	MAE	MSE	RMSE	MAPE
BP	0.80719	5.6252	42.2318	6.4986	16.159%
GA-BP	0.88471	3.6312	19.1067	4.3711	10.0244%

**Table 4:** The 28d compressive strength test results and predictions.

W/B	CC kg/m^3^	S kg/m^3^	G kg/m^3^	FA kg/m^3^	W kg/m^3^	WR	fce MPa		
							Measured Value	Predicted Value	
								BP	GA-BP
0.57	238	752	1128	122	205.2	7.9	33.2	40.1	33.2
0.57	204	752	1128	163	209.19	8.1	31.9	37.8	33.8
0.57	170	752	1128	204	213.18	8.2	32.5	36.1	32.4
0.57	136	752	1128	245	217.17	8.4	35.4	34.8	32.3
0.5	340	846	1034	0	170	0.5	31.9	34.1	33.4
0.5	238	846	1034	122	180	0.52	32.8	43.1	28.8
0.5	204	846	1034	163	183.5	0.5	33.7	41.2	31.5
0.5	170	846	1034	204	187	0.49	35.2	42.4	31.5
0.5	136	846	1034	245	190.5	0.49	36.8	44.5	31.4
0.62	340	940	940	0	210.8	7.5	37.4	47.2	31.1

**Table 5:** Carbon emission factors and price for building materials.

Type of Building Material	Prices (CNY/kg)	EF_i_
X1	0.442	735 kg CO_2_e/t
X2	0.136	2.51 kg CO_2_e/t
X3	0.102	2.18 kg CO_2_e/t
X4	0.369	8.77kg CO_2_e/t
X5	0.002	0.168 kg CO_2_e/t
X6	4.75	0.0285 kg CO_2_e/kg

**Table 6:** NSGA-II optimization parameters and results.

NO.	X1 kg/m^3^	X2 kg/m^3^	X3 kg/m^3^	X4 kg/m^3^	X5 kg/m^3^	X6	fce MPa	Cost CNY	CE kg CO_2_e/m^3^
1	331.3	639.4	1039	56	153	0.632	42.7	363.3	243.9
2	445	816.1	1113	71	154	6.27	68	447.7	327.8
⋮	⋮	⋮	⋮	⋮	⋮	⋮	⋮	⋮	⋮
39	337.6	635.2	1103	88	154	7.442	65.6	431.5	270.8
40	331.3	639.4	1039	56	154	1.095	43.6	365.5	244.0

**Table 7:** Comparison of experimental and predicted values of 28d compressive strength of concrete.

Material Consumption/m^3^		fce/MPa			Cost/CNY			CE/kg CO_2_e		
		Measured Value	Predicted Value	RE	Measured Value	Predicted Value	RE	Measured Value	Predicted Value	RE
X1/Kg	331.3	52.6	51.5	2.1%	387.82	385.5	0.6%	282.45	274.37	2.9%
X2/Kg	639.4									
X3/Kg	1039									
X4/Kg	56									
X5/Kg	153									
X6/kg	0.632									

**Table 8:** Main chemical compositions of cement.

Chemical Composition	MgO	Al_2_O_3_	SiO_2_	SO_3_	CaO	Fe_2_O_3_	Loss
Mass fraction	1.4	4.96	18.76	1.43	57.56	33.352	---

**Table 9:** Comparison of concrete strength, cost, and carbon emissions.

Type	X1 kg	X2 kg	X3 kg	X4 kg	X5 kg	X6 kg	Cost CNY	CE kg CO_2_e	fce MPa
This scheme	331.3	639.4	1039	56	153	0.632	387.82	282.45	52.6
C_50_	451	574.8	1193.8	0	180.4	3.38	418.07	335.66	57.75

## Data Availability

The original contributions presented in the study are included in the article, further inquiries can be directed to the corresponding author.
